# PRL2 negatively regulates FcεRI mediated activation of mast cells

**DOI:** 10.1038/s41419-025-07649-2

**Published:** 2025-04-21

**Authors:** Xin Guo, Yunxuan Lei, Yanhua Xu, Xinyue Du, Lin Lin, Yanping Luo, Yebin Xi, Yinshi Guo, Xiaoyin Niu, Zhaojun Wang, Guangjie Chen

**Affiliations:** 1 Department of Immunology and Microbiology, Shanghai Institute of Immunology, Shanghai Jiao Tong University School of Medicine, Shanghai, China; 2 Department of Allergy, Ren Ji Hospital, Shanghai Jiao Tong University School of Medicine, Shanghai, China; 3 Department of Laboratory Medicine, Ruijin Hospital, Shanghai Jiao Tong University School of Medicine, Shanghai, China

**Keywords:** Immunology, Inflammation

## Abstract

Mast cells play a central role in allergic reactions, acting as key effector cells that initiate and amplify the inflammatory response. In this study, we demonstrate that phosphatase of regenerating liver 2 (PRL2) functions as a negative regulator of FcεRI-mediated mast cell activation. In PRL2-deficient myeloid cells, PRL2 conditional knockout mice developed more severe passive systemic anaphylaxis (PSA). Although PRL2 deficiency does not impact mast cell development, in the absence of PRL2 FcεRI-mediated mast cell activation is enhanced. In the presence of IgE the expression of mast cell PRL2 is downregulated, leading to modulation of the cellular response. In PRL2-deficient mast cells, the PI3K signaling pathway is upregulated, resulting in increased calcium influx. This, in turn, enhances mast cell degranulation and the production of inflammatory mediators. Moreover, hydroxychloroquine (an inhibitor of PRL2 degradation) reduces the severity of PSA in wild-type mice. Our findings suggest that PRL2 acts as a negative regulator of FcεRI-mediated mast cell activation. Therefore, therapeutic strategies aimed at enhancing PRL2 activity in mast cells may offer a promising approach for the treatment of allergic disorders.

## Introduction

Passive systemic anaphylaxis (PSA) represents a critical immune-mediated reaction characterized by severe, rapid-onset, and potentially life-threatening symptoms. This condition occurs when preformed IgE antibodies, specific to a particular allergen, bind to high-affinity IgE receptors (FcεRI) on mast cells (MCs) and basophils throughout the body. Subsequent re-exposure to the same allergen triggers the cross-linking of these IgE-FcεRI complexes, leading to the degranulation of MCs and basophils. PSA is often studied in animal models, particularly in mice, to understand the mechanisms underlying allergic reactions and to develop therapeutic interventions [1].

MCs are tissue-resident cells and uniquely required for immediate hypersensitivity. MCs play a pivotal role in allergic reactions, serving as key effector cells responsible for initiating and amplifying the inflammatory response [1]. Their strategic localization in tissues exposed to the external environment, such as skin, lungs, and mucous membranes, ideally positions them to rapidly respond to allergens. Upon allergen encounter, IgE-sensitized MCs undergo activation, leading to the release of a myriad of inflammatory mediators including histamine, proteases, leukotrienes, prostaglandins, and cytokines [2–4]. These mediators orchestrate the clinical manifestations of allergic responses, ranging from localized symptoms such as sneezing, itching, and rhinorrhea to systemic anaphylactic reactions characterized by widespread vasodilation, bronchoconstriction, hypotension, and even shock. Although basophils and eosinophils also contribute to allergic inflammation, their roles are generally considered less central compared to MCs. Because of the latter’s abundance in peripheral tissues, rapid response to allergen challenge, and ability to release a broad spectrum of inflammatory mediators [5].

Phosphatase of regenerating liver 2 (PRL2), encoded by the protein tyrosine phosphatase 4A2 gene, is a member of the PRL phosphatase family [6]. It plays crucial roles in cell proliferation, survival, migration, and adhesion, processes closely linked to tumorigenesis. PRL2 is frequently overexpressed in various human tumors and promotes cancer progression [6, 7]. However, its role is context-dependent, exhibiting dualistic functions in cancer biology. For example, PRL2 is upregulated in 100% of hepatocellular carcinomas but downregulated in 54% of renal cancers [8]. These findings suggest that PRL2 may play pleiotropic roles in tumorigenesis, acting as a driver of tumor progression and a marker of aggressiveness in certain tissues, while potentially suppressing tumor formation or growth in others.

Moreover, it is also implicated in immune regulation and inflammatory responses. Our previous studies revealed that PRL2 is highly expressed in innate immune cells and can regulate inflammation and antibacterial immune responses [9, 10]. We also revealed the role of PRL2 in neutrophil extracellular trap (NET) formation, which is critical in diseases such as severe malaria and acute lung injury. By regulating the Rac-ROS pathway, PRL2 modulates neutrophil activation and NET accumulation, thereby contributing to disease progression [11].

Although PRL2 is highly expressed in MCs [12] (expression pattern of *Ptp4a2* in MCs from BioGPS database) any potential role it might play in allergic disease remains unclear. Because we found that PRL2 myeloid conditional knockout (KO) mice exhibit heightened susceptibility to PSA we hypothesized that PRL2 may directly participate in regulating MCs and affect their function in allergic disease. In this study, we demonstrate that PRL2 functions as an inhibitory factor in FcεRI-mediated MC activation. In vitro experiments revealed that bone marrow-derived MCs (BMMCs) lacking PRL2 developed normally. However, in response to IgE-mediated activation they exhibited enhanced calcium influx, degranulation, and mediator release. PRL2 modulates MC activation and degranulation by influencing the PI3K pathway. Notably, the use of hydroxychloroquine (HCQ), which inhibits PRL2 degradation, alleviates PSA symptoms in WT mice. Our results indicate that PRL2 plays a crucial role in suppressing FcεRI-mediated MC activation and allergic disease, suggesting that targeting PRL2 could represent a promising therapeutic strategy for treating allergies.

## Materials and methods

### Animal models

Male C57BL/6 PRL2 myeloid conditional KO mice (*Ptp4a2*^*fl/fl*^*LysM*^*Cre+*^) (CKO) aged 6–10 weeks were provided by Professor Zhaojun Wang (Shanghai Jiao Tong University School of Medicine), and mice were generated as previously described [10]. Wild-type (WT) male C57BL/6 were purchased from Shanghai Lingchang Biotechnology Company Ltd. The sample size for animal experiments was determined through power analysis and adjusted according to previous studies. Subsequently, animals were randomized into different groups. The mice were maintained under specific pathogen-free conditions and provided autoclaved food and water. All animal experiments were approved by the Institutional Animal Care and Use Committee (IACUC) of Shanghai Jiao Tong University School of Medicine (project number: A-2019-053, 069). There was no blinding of researchers or participants.

### Passive systemic anaphylaxis

Mice were intravenously (*i.v*.) injected with 10 μg of anti-2,4-dinitrophenol (DNP) mouse IgE antibody (SPE-7; Sigma-Aldrich) in 200 μL sterile PBS to induce PSA. The following day, mice were sensitized via an intravenous injection of 100 μg of DNP-bovine serum albumin (DNP-BSA, LGC Biosearch Technologies, Petaluma, Calif, USA) in 100 μL of sterile PBS. Body temperature was measured every 5 min for 100 min using a rectal thermometer. After 100 min, mice were euthanized by intraperitoneal injection of pentobarbital sodium, and blood samples were collected to measure the serum concentration of MC protease-1 (MCPT-1), leukotriene C4 (LTC4), prostaglandin D2 (PGD2) and histamine. Lung tissues were collected for paraffin embedding and HE staining to detect pulmonary vascular extravasation.

For HCQ treatment, mice were given a dose of 80 mg/kg/d of HCQ (SPH Zhongxi Pharmaceutical Co., Ltd, China) by oral gavage a week before the PSA model was performed. The control group received an equal volume of normal saline (NS) instead of HCQ.

### Proteomics

Cultured mature BMMCs were incubated overnight with anti-DNP IgE followed by stimulation with 50 ng/mL DNP-BSA for 2 h. The cells were then prepared for further protease analyses. The samples were denatured with 2% SDS buffer containing 50 mM. DTT and boiled at 100 °C. The proteins were then alkylated using iodoacetamide and subjected to protein precipitation using precooled acetone. Sequencing-grade modified trypsin (Promega) was used to enzymatically digest the protein. The resulting peptides were collected by centrifugation, followed by the purification with C18 Zedtips and elution with acetonitrile containing 0.1% TFA. The peptides were then vacuum-concentrated and dried using SpeedVac (ThermoSavant). iRT peptides (Biognosys AG, Switzerland) were added, according to the manufacturer’s instructions, to the resuspended peptides prior to mass spectrometry (Orbitrap Exploris 480, Thermo Fischer Scientific, MA, USA). The mass spectrometer was run in DIA data acquisition mode using a mixed data strategy and the raw data processed and analyzed by Spectronaut 14 software (Biognosis AG, Switzerland) with default settings. The false discovery rate for precursor ion and protein levels was set to 1% and global standardization employed. Sequences were analyzed using the UniProt mouse database. Significant peptides (*P* < 0.05, FC > 1.2) were grouped into clusters and further analyzed.

### Bone marrow-derived mast cells

Bone marrow cells were obtained by flushing the bone marrow from the femurs and tibias of mice. Cells were cultured in RPMI 1640 medium (Thermo Fisher Scientific) with 10% fetal bovine serum (FBS; Gibco), 2 mM L-glutamine, 100 U/mL penicillin, 100 μg/mL streptomycin, 10 mM HEPES, 1 mM sodium pyruvate, 0.1 mM nonessential amino acids and 55 μM 2-mercaptoethanol (all from Thermo Fisher Scientific). The culture medium also contained 10 ng/mL IL-3 and 20 ng/mL SCF (both from Peprotech) to induce the differentiation of bone marrow derived mast cells (BMMCs). The culture medium was changed every 3 days. After 4–6 weeks of culture BMMCs with a purity of >95%, defined by co-expression of c-kit and FcεRI in flow cytometry, were used for subsequent experiments.

### Transfection and infection

Stable cell lines with PRL2 overexpression were created by retrovirus infection. Packaging cells (293T) were transfected with MSCV-based retrovirus vector MigR1 and pVSVG and pCGP sequences using Attractene to produce retroviruses. After 24–36 h, the culture supernatants were collected and filtered. Immature KO BMMCs were infected with the filtered viral supernatants in the presence of 5 μg/ml polybrene (Sigma-Aldrich, USA) for 12 h, after which the medium was changed. Cells infected with MigR1 empty virus were used as control cell line. After infection, cells were cultured until mature and analyzed using Western blot before proceeding with experimental applications.

### MC degranulation assay

BMMCs were incubated overnight at 37 °C with 1 μg/mL anti-DNP IgE antibody (SPE-7; Sigma-Aldrich) and stimulated with 50 ng/mL DNP-BSA antigen in 100 μL of Tyrode buffer (Procell). The culture supernatant (50 μL) was collected. Cell lysates were incubated with Triton X-100 in Tyrode buffer, and 50 μL of the cell lysate supernatant was collected in a new plate well. Subsequently, both supernatants were incubated at 37 °C for 1 h with 100 μL of P-nitrophenyl-N-acetyl-D-glucosamide (1.3 mg/ml in 0.1 M citrate, pH 4.5) (Sigma-Aldrich). The enzyme reaction was stopped using 50 μL of 0.5 M glycine (pH 10.6; Shanghai Yuanye Bio-Technology Co., Ltd). Absorbance was measured at 405 nm using a microplate reader (Bio-Rad).

For inhibitor treatment, IgE-sensitized BMMCs were pretreated with 100 nM wortmannin (MCE, HY-10197) for 2 h before stimulation with DNP-BSA.

### Ca^2+^ measurement

BMMCs were suspended overnight in culture medium containing 1 μg/mL anti-DNP-IgE. Cells were washed twice with pre-warmed PBS and loaded with Fluo-8 AM (Abcam, Cambridge, UK, ab142773, 3 µM) for 40 min at 37 °C. The cells (1 × 10^6^) were washed with pre-warmed Tyrode buffer, stimulated with 50 ng/mL DNP-BSA, and analyzed using time-lapse flow cytometry [13] (BD Biosciences). Additionally, the cells (5 × 10^4^) were washed with pre-warmed Tyrode buffer and incubated in a poly-D-lysine (Sigma-Aldrich)-treated 8-well Chambered Coverglass (Thermo Fisher Scientific) for 20 min. Intracellular calcium flux was induced with DNP-BSA (Biosearch Technologies) treatment, and fluorescence emission monitored using a confocal microscope [14] (Leica SP8). Data were analyzed using Fiji software.

For inhibitor treatment, IgE-sensitized BMMCs were pretreated with 100 nM wortmannin (MCE, HY-10197) for 2 h before stimulation with DNP-BSA.

### Flow cytometry analysis of MC degranulation

Briefly, BMMCs were incubated with anti-DNP-IgE as previously described [13]. Baseline fluorescence was recorded for 2 min and the cells were then stimulated with DNP-BSA in the presence of Avidin-Sulforhodamine 101 (Av.SRho) for 20 min. Data were analyzed using the FlowJo software.

### Single-cell analysis of MC degranulation

Degranulation was analyzed using confocal microscope as previously described [15]. Briefly, IgE-sensitized BMMCs (5 × 10^4^) were loaded with Fluo-8 AM and placed into poly-D-lysine (Sigma-Aldrich) treated 8-well chambered cover glass (Thermo Fisher Scientific) for 20 min at 37 °C. Baseline fluorescence was recorded for 2 min. DNP-BSA was added in the presence of Av.SRho. Data were recorded with minimal time intervals for a total of 30 min in a controlled atmosphere using a Leica SP8 confocal microscope. 63×/1.40 oil objective and electric zoom 2 (8 bits/pixel 512 × 512) were used for high-resolution single-cell analysis. The mean fluorescence intensity was quantified using the measurement function of the Fiji software.

### 3-D degranulation assay

IgE-sensitized BMMCs were placed into poly-D-lysine (Sigma-Aldrich)-treated 8 well-chambered cover glass (Thermo Fisher Scientific) for 20 min at 37 °C. DNP-BSA was added to the cells in Tyrode buffer supplemented with Av.SRho. After 30 min following stimulation, Z-stack image sequences of single BMMC were acquired immediately in 3-D up to a depth of 20–30 μm using 63×/1.40 oil immersion objective lens and electric zoom of 5 (8 bits/pixel 1024×1024). Modeling and analysis of the released granules were performed using the Imaris Bitplane software.

### Immunofluorescence staining

Lungs were fixed with 4% paraformaldehyde (PFA; Servicebio) overnight at room temperature. And then 4–5 μm paraffin sections of mouse lung tissue were prepared for immunofluorescence staining. Following deparaffinization and rehydration, the sections were immersed in citrate antigen retrieval solution (pH 6.0) and maintained at a sub-boiling temperature for 8 min. After standing for an additional 8 min without heating the sections were subjected to another sub-boiling treatment for 4 min. To permeabilize the sections, 0.5% Triton X-100 (Solarbio) was applied for 30 min, followed by blocking with 20% goat serum (Servicebio) for 2 h at room temperature. The tissue sections were then incubated with the primary antibody overnight at 4 °C in a humidified chamber. After washing, the sections were incubated with secondary antibodies for 2 h at room temperature. Finally, the sections were mounted using DAPI Fluoromount (Yeasen) and visualized with a Leica SP8 confocal microscope. The primary antibodies used were: mouse anti-MC tryptase (Abcam, ab2378, [AA1], 1:500), rabbit anti-PRL2 (Novus, NBP2-93937, 1:200). The secondary antibodies used were: Alexa Fluor647 anti-mouse IgG (CST, 4410,1:1000), Alexa Fluor594 anti-rabbit IgG (CST, 8889,1:1000).

### RNA isolation and qPCR

Total RNA was extracted from cultured BMMCs using the RNAiso Plus reagent (Takara Bio). cDNA was generated using Hifair III 1st Strand cDNA Synthesis SuperMix for qPCR (Yeasen) and amplified using Hieff qPCR SYBR Green Master Mix (Yeasen) along with specific primer pairs using the ViiA 7 Real-time PCR system (Applied Biosystems). The expression of each gene was normalized to that of *Actb*. The primer sequences used in this study are listed in Table S1.

### Enzyme-linked immunosorbent assay (ELISA)

Cytokine production was determined using mouse IL-4, IL-6, TNF-α, CCL2 and MCPT-1 ELISA kits (Thermo Scientific, 88-7044-22, 88-7064-88, 88-7324-88, 88-7391-22 and 88-7503-22, respectively), mouse IL-9, IL-13, GM-CSF, CCL1, and CCL3 ELISA kits (Jianglaibio, JL20272, JL20247, JL12479, JL42145, JL20414, respectively), leukotriene C4 and prostaglandin D2-MOX ELISA kits (Cayman Chemical, 501070, 512011, respectively), and histamine ELISA kit (Labor Diagnostika Nord, BA E-5800R), following the manufacturer’s instructions.

### Western blot

BMMCs were centrifuged, washed with PBS, and lysed in radioimmunoprecipitation assay buffer containing a protease and phosphatase inhibitor cocktail (Beyotime Biotechnology). Samples were mixed with 6× protein-loading buffer (TransGen Biotech) and boiled for 10 min. Proteins were resolved by SDS-PAGE and transferred onto polyvinylidene fluoride membranes (Millipore, USA). The membranes were blocked with 5% milk for 1 h at room temperature. Primary antibodies were incubated overnight at 4 °C and secondary antibodies incubated for 1 h at room temperature. Signals were detected using a western chemiluminescent HRP substrate (Millipore) and the bands analyzed using ImageJ softmare. The following primary antibodies were used: mouse anti-PRL2 (Millipore, 05-1583, clone 42, 1:1000), rabbit anti-β-actin (CST, 4970, 13E5, 1:1000), rabbit anti-phospho-PLCγ1 (CST, 14008, [D6M9S], 1:1000), rabbit anti-phospho-PI3 kinase p85/p55 antibody (CST, 4228, 1:1000), rabbit anti-PI3 kinase p85 antibody (CST, 4257, [19H8], 1:1000), rabbit anti-PLCγ1 (CST, 5690, [D9H10], 1:1000), rabbit anti-VAMP-8 (Proteintech, 15546-1-AP, 1:1000), rabbit anti-SNAP-23 (Proteintech, 10825-1-AP, 1:1000). The following secondary antibodies were used: HRP-linked anti-rabbit IgG (CST, 7074, 1:2000), and HRP-linked anti-mouse IgG (H + L) (Jackson Immunoresearch, 115-035-003, 1:2000), HRP-linked anti-rabbit IgG, light chain specific (Jackson Immunoresearch, 211-032-171, 1:10,000).

### Co-immunoprecipitation (Co-IP)

IgE-primed WT and KO BMMCs were stimulated with DNP-BSA for indicated time. Cells were lysed with Lysis Buffer (Epizyme Biotech, China) supplemented with protease and phosphatase inhibitor cocktail (Beyotime, China). The extracts were assayed for protein concentration. Co-IP was performed using rProtein A/G Agarose Resin (Yeasen, China). In brief, an equal amount of cell lysate was incubated with SNAP-23 antibodies overnight at 4 °C with rotation. rProtein A/G Agarose Resin were incubated with protein-antibody complex for 3 h at 4 °C with rotation. The captured Protein A/G/Ab/Ag complex was washed with Lysis Buffer twice and boiled in 2× loading buffer. Then SDS-PAGE and western blot were performed to detect the protein.

### Histology

Mouse tissues were fixed with 4% PFA at room temperature overnight, embedded in paraffin, and cut into 4–5 μm sections. The slices were stained with H&E and images captured using a BX53 microscope (Olympus Corporation, Tokyo, Japan).

### Statistical analysis

Data analysis was conducted using GraphPad Prism and the results are reported as the mean ± SEM. Statistical significance was determined using a two-tailed unpaired student’s *t* test with Welch correction or Mann–Whitney U test between two groups. Two-way ANOVA or Kruskal–Wallis test was employed for comparisons between multiple groups. Statistical significance was set at *P* < 0.05.

## Results

### PRL2 deficiency exacerbates passive systemic anaphylaxis (PSA) in vivo

**Fig. 1:**
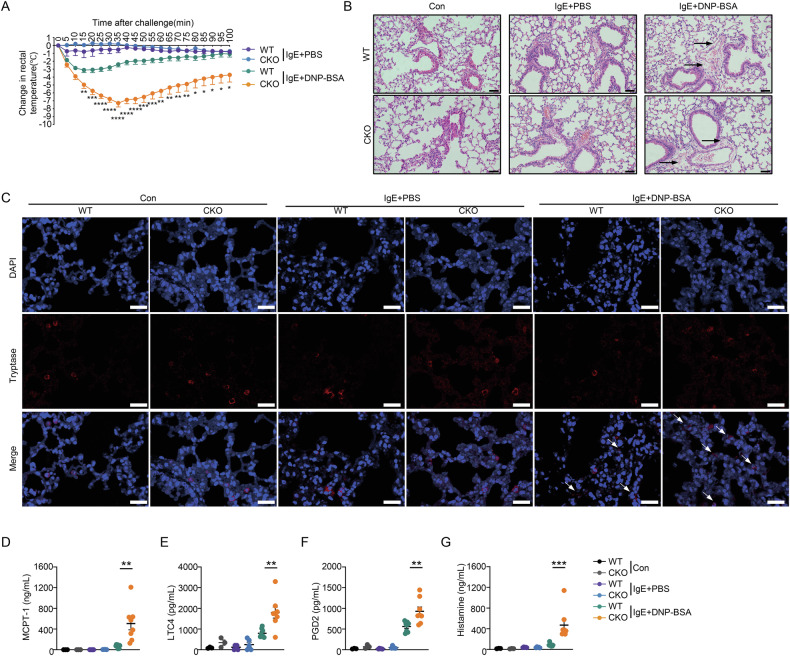
PRL2 deficiency aggravates PSA in vivo. **A** Mice were injected intravenously with 10 μg anti-DNP-IgE per mouse overnight and challenged with PBS or 100 μg DNP-BSA. Rectal temperatures of mice were assessed every 5 min (*n* = 3 for Con group, *n* = 5 for IgE+PBS group, *n* = 8 mice for IgE+DNP-BSA group, ^*^P < 0.05, ^**^P < 0.01, ^***^P < 0.005, ^****^P < 0.0001). **B** Representative images of pulmonary perivascular edema from control (Con) mice and in the indicated experimental groups 100 min post-challenge (black arrow: pulmonary perivascular edema). Scale bar, 50 μm. **C** Representative images of mast cells with tryptase staining from Con mice and in the indicated experimental groups 100 min post-challenge (white arrow: degranulated mast cell). Scale bar, 25 μm. Serum levels of MCPT-1 (**D**), LTC4 (**E**), PGD2 (**F**), and histamine (**G**) from mice were determined by ELISA (*P* = 0.0031 for MCPT-1, *P* = 0.0027 for LTC4, *P* = 0.0062 for PGD2, *P* = 0.0002 for histamine). Data were obtained from two independent experiments, and individual values are shown in circles. Data are presented as the mean ± SEM and analyzed using two-way ANOVA (**A**), two-tailed unpaired *t* test (**D**–**F**), and two-tailed Mann–Whitney U test (**G**). ^*^P < 0.05, ^**^P < 0.01, ^***^P < 0.005, ^****^P < 0.0001.

We conducted in vivo experiments using WT and CKO mice to explore the role of PRL2 in MC function. PSA, an IgE-dependent type I immediate hypersensitivity reaction, was assessed. Rectal temperature was recorded to measure the magnitude of the PSA model. Within 35 min, the CKO mice showed a more progressive decrease in temperature compared with WT mice. (Fig. 1A). Histologic analysis of the lung between PSA groups showed pulmonary perivascular edema, a hallmark of airway anaphylactic reactions, was exacerbated in CKO mice compared to the WT mice (Fig. 1B). Meanwhile, the number of degranulated cells that with fuzzy outline and diffuse staining around cells was greater in lung tissue from CKO mice (Fig. 1C). Furthermore, the release of MCPT-1, LTC4, PGD2 and histamine in response to anaphylaxis was higher in CKO mice than in WT mice (Fig. 1D–G). The proportion of lymphocytes, monocytes, neutrophils, eosinophils, and basophils in peripheral blood was comparable between WT and CKO mice (Fig. S1). Overall, these findings suggest that PRL2 deficiency promotes IgE-FcεRI-mediated reactions and pathology in vivo thereby enhancing systemic anaphylactic responses.

### PRL2 deficiency controls FcεRI-mediated MC degranulation and cytokine expression

**Fig. 2:**
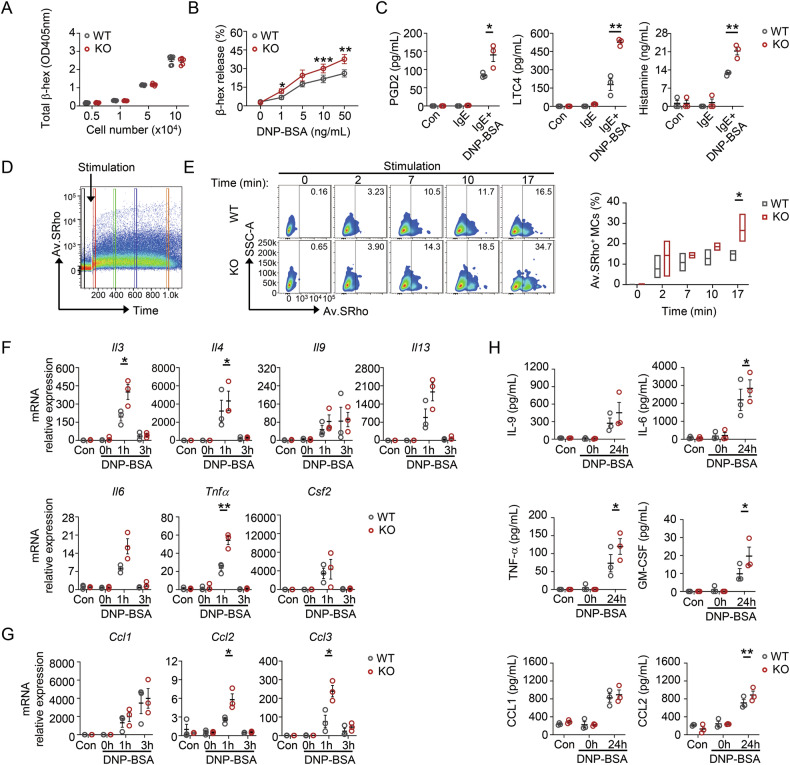
PRL2 deficiency promotes IgE-mediated degranulation and cytokine expression. **A** The total content of β-hexosaminidase in the BMMCs was examined by measuring the absorbance of cell lysates at OD405 nm (*n* = 6 for each group). **B**–**H** WT and KO BMMCs were sensitized with anti-DNP-IgE and stimulated with DNP-BSA for indicated time. **B** BMMCs were activated with indicated concentrations of DNP-BSA for 30 min and dose-dependent β-hexosaminidase release was measured (*n* = 6 for each group, ^*^P = 0.0158, ^**^P = 0.0078, ^***^P = 0.0008). **C** Degranulation was assessed by measuring the production of PGD2, LTC4, and histamine at 6 h (*n* = 3 for each group, *P* = 0.0409 for PGD2, *P* = 0.0023 for LTC4, *P* = 0.0051 for histamine). **D** Av.SRho staining kinetics after stimulation were analyzed by flow cytometry. The arrow indicates the time of DNP–BSA addition to the cells. **E** Representative time-lapse degranulation (left panel) and frequency of Av.SRho^+^ BMMCs (right panel) upon DNP-BSA stimulation for indicated time using flow cytometry (*n* = 3 for each group, *P* = 0.0374). Cytokine (**F**) and chemokine (**G**) gene expression of BMMCs stimulated with 50 ng/mL DNP-BSA for indicated time were analyzed by qPCR, normalized to WT control (Con) group (*n* = 3 for each group, *P* = 0.0313 for *Il3*, *P* = 0.0345 for *Il4*, *P* = 0.0039 for *Tnfα*, *P* = 0.0289 for *Ccl2*, *P* = 0.0234 for *Ccl3*). **H** The production of cytokine and chemokine from (**F**) and (**G**) were measured by ELISA at 24 h with DNP-BSA stimulation (*n* = 3 for each group, *P* = 0.0391 for IL-6, *P* = 0.0186 for TNF-α, *P* = 0.0432 for GM-CSF, *P* = 0.0095 for CCL2). Data were obtained from three (**A**–**C**, **E**–**H**) independent experiments. Data are presented as the mean ± SEM and analyzed using two-tailed unpaired *t* test. ^*^P < 0.05, ^**^P < 0.01, ^***^P < 0.001.

After culturing for 4–6 weeks in the presence of IL-3 and SCF, mature BMMCs were obtained for subsequent experiments (Fig. S2A). PRL2 expression was measured in both WT and KO BMMCs and Western blot analysis showed a reduction of PRL2 in KO BMMCs (Fig. S2B). The proliferation of WT and KO BMMCs was comparable (Fig. S2C). β-hexosaminidase exists in all types of granules in MCs [16], and the total contents in the BMMCs, reflected by measuring the absorbance of cell lysates at OD405nm showed no difference (Fig. 2A). However, the loss of PRL2 promoted BMMC degranulation. IgE-primed WT and KO BMMCs were stimulated with DNP-BSA to explore the physiological role of PRL2 in anaphylactic reactions. The release of β-hexosaminidase was higher in KO BMMCs than in WT BMMCs (Fig. 2B). In accordance with this result, PRL2 deficiency resulted in elevated IgE-FcεRI-elicited release of PGD2, LTC4 and histamine (Fig. 2C). We then used flow cytometry to measure the extent of degranulation of individual cells at the population level using fluorescent avidin-SRho (Fig. 2D). Upon stimulation, the KO BMMCs showed a higher proportion of cells that underwent degranulation (Fig. 2E and Video. S1). Consistent with this result, the gene expression of pro-inflammatory cytokines and chemokines increased in KO BMMCs upon stimulation with DNP-BSA (Fig. 2F, G). Moreover, KO BMMCs exhibited a remarkably higher secretion of the cytokines IL-6, TNF-α, GM-CSF, and CCL2 than the WT BMMCs (Fig. 2H). The levels of IL-4, IL-13, and CCL3 were below the detection limit of the kit, and thus these data were not included. Additionally, the presence of IL-3 in the culture medium interfered with the precise quantification of release levels following cell activation. Besides, we also observed the attenuated degranulation rate and cytokines expression in KO BMMCs following overexpression of PRL2 (Fig. S3). Hence, PRL2 deficiency negatively regulates FcεRI-mediated MC degranulation and cytokine expression.

### PRL2 deficiency affects calcium signaling and degranulation dynamics

**Fig. 3:**
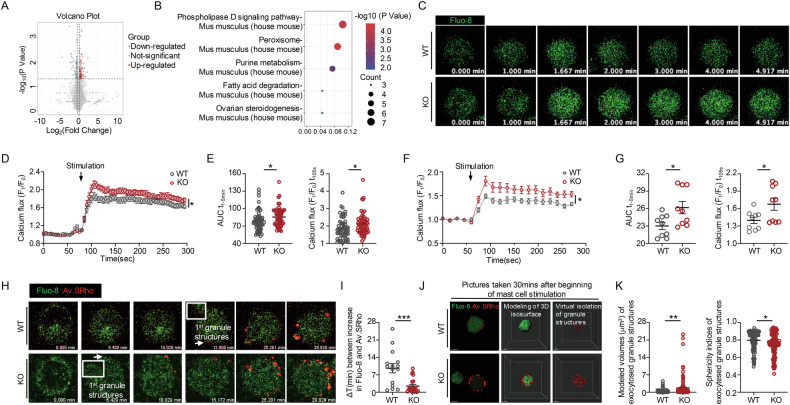
PRL2 deficiency increases FcεRI-mediated calcium and degranulation dynamics. **A**, **B** WT and KO BMMCs were sensitized with anti-DNP-IgE and activated with DNP-BSA for 2 h, then protease analysis was performed. (**A**) Volcano plot depicting the 168 differentially expressed proteins in KO BMMCs with fold change threshold of 1.2 (|FC| > 1.2) and *P* < 0.05. **B** KEGG pathway analysis of differentially expressed proteins showed five enriched terms. **C** Fluo-8 AM dependent measurements of cytosolic Ca^2+^ concentrations in the Tyrode buffer. BMMCs were sensitized with anti-DNP-IgE and DNP-BSA was added at 1 min, data was recorded by time-lapse confocal microscope for 5 min. Representative time-lapse of a single BMMC. The time scale reflects the kinetics of the calcium responses (Green, [Ca^2+^]_i_). **D** WT and KO BMMCs were pretreated with DNP-specific IgE antibodies (1 μg/mL) overnight and stimulated with DNP-BSA (50 ng/mL) at 1 min (arrow). Data was normalized to the initial F_1_/F_0_ ratio (*n* = 55 cells for WT group and *n* = 56 cells for KO group, *P* = 0.04). **E** Quantitative AUC (left panel) was used to estimate the overall increase in cytoplasmic Ca^2+^ concentration over time and histogram (right panel) showing average peak cytoplasmic Ca^2+^ concentration at 105 s from (**D**) (*n* = 55 cells for WT group and *n* = 56 cells for KO group, *P* = 0.0252 for AUC t_1–5 min_, *P* = 0.0153 for Calcium flux (F_1_/F_0_) t_105s_). **F** Fluo-8 AM dependent measurements of cytosolic Ca^2+^ concentrations in the Tyrode buffer using time-lapse flow cytometry. WT and KO BMMCs were pretreated with DNP-specific IgE antibodies (1 μg/mL) overnight and stimulated with DNP-BSA (50 ng/mL) at 1 min (arrow). Data was normalized to the initial F_1_/F_0_ ratio (*n* = 9 for each group, *P* = 0.0202). **G** Quantitative AUC (left panel) was used to estimate the overall increase in cytoplasmic Ca^2+^ concentration over time and histogram (right panel) showing average peak cytoplasmic Ca^2+^ concentration at 105 s from (**F**) (*n* = 9 for each group, *P* = 0.0216 for AUC t_1–5 min_, *P* = 0.0294 for Calcium flux (F_1_/F_0_) t_105s_). **H** Representative time-lapse of a single Fluo-8 (green, [Ca^2+^]_i_)-labeled BMMC activated with DNP-BSA in the presence of Av.SRho (red, identifying exteriorized granule structures). White insets show a budding granule structure at higher magnification. Arrows indicate the first budding granule structures; the time scale reflects the kinetics of the responses induced by DNP-BSA. **I** Mean ΔT between the calcium increase and the first detection of the budding structure following DNP-BSA stimulation from (**H**) was measured (*n* = 15 cells for the WT group and *n* = 22 cells for the KO group, *P* = 0.0003). **J** 3D photographs of single BMMCs 30 min after exposure to DNP-BSA. WT BMMCs (upper panels), KO BMMCs (lower panels). Panels, left to right: Av.SRho (red) merged with Fluo-8 (green); iso surface modeling of Av.SRho (red) and Fluo-8 (green); virtual isolation of granule structures. **K** Modeled volumes (in cubic micrometers, assessing granule structures) (*n* = 111 for the WT group and *n* = 104 for the KO group, *P* = 0.006). Modeled sphericity indices (from 0 to 1, 1 being a perfect sphere) (*n* = 111 for the WT group and *n* = 104 for the KO group, *P* = 0.0191). Data were obtained from five (**D**, **E**), three (**F**, **G**) and four (**I**, **K**) independent experiments. Confocal images (**H**, **J**) are representative of four experiments. Values in the bar graphs are presented as the mean ± SEM and analyzed via two-way ANOVA (**D**, **F**), two-tailed unpaired *t* test (**G**), and two-tailed Mann–Whitney U test (**E**, **I**, **K**). ^*^P < 0.05, ^**^P < 0.01, ^***^P < 0.001.

We performed proteomic analysis on antigen-activated BMMCs to investigate the potential properties of PRL2 in MCs. We defined 8297 proteins after matching the peptides to proteins using the UniProt database. The differentially expressed genes were identified using volcano plots (Fig. 3A). The signaling pathway analysis based on the Kyoto Encyclopedia of Genes and Genomes (KEGG) database showed that the phospholipase D signaling pathway, involved in a variety of cellular processes [17], was most enriched (Fig. 3B). PLD signaling mediates an increase in calcium signaling, which plays a role in MC activation [18]. Calcium mobilization is the key regulator of MC degranulation [19, 20]. Next, we loaded BMMCs with Fluo-8 AM and stimulated them with DNP-BSA. KO BMMCs showed an increased and sustained cytosolic Ca^2+^ influx compared to their WT counterparts (Fig. 3C–E and Video. S2). Accordingly, flow cytometry measurements of changes in calcium concentration in WT and KO BMMCs with DNP-BSA stimulation showed the same trend towards increased calcium influx in KO BMMCs (Fig. 3F, G). Next, we utilized the Av.SRho probe to capture the exteriorized secretory granules to investigate the spatiotemporal features of MC degranulation [15]. We loaded Fluo-8 AM into anti-DNP-IgE sensitized BMMCs and stimulated them in the presence of Av.SRho to monitor early [Ca^2+^]_i_ and degranulation dynamics in single cells and real-time using high-resolution confocal microscopy. DNP-BSA-mediated BMMC stimulation led to earlier detection of the first budding granule in KO BMMCs compared to WT BMMCs (Fig. 3H and Video. S3). Additionally, WT BMMCs exhibited a lag time (ΔT) of ~8 min between the early increase in [Ca^2+^]_i_ and the detection of the first Av.SRho^+^ exteriorized granule structures. This ΔT was shortened in KO BMMCs (Fig. 3I), indicating a quicker activation of MCs with PRL2 deficiency. We employed a 3D degranulation assay to virtually isolate the exteriorized granules to further analyze the physical characteristics of the released granule structures [15]. Computational 3D analysis revealed that these granule structures were larger and exhibited heterogeneous shapes (Fig. 3J, K). Hence, PRL2 deficiency may lead to enhanced FcεRI-mediated MC activation by upregulating calcium influx. Additionally, KO BMMCs exhibited distinct secretory strategies when stimulated by DNP-BSA and PRL2 deficiency led to the exteriorization of secretory granule structures with varying physical characteristics. Given the critical role of SNARE proteins in mediating the exocytotic mechanism of MCs [15, 21] and their potential regulatory link to PRL2, we conducted co-immunoprecipitation (Co-IP) assays to investigate SNARE complex formation under antigen stimulation. In the resting state, a subtle interaction between VAMP-8 and SNAP-23 was observed in PRL2-KO cells. Upon DNP-BSA stimulation, the formation of the VAMP-8 and SNAP-23 complex increased in PRL2-KO cells (Fig. S4).

### PRL2-deficiency affects IgE-FcεRI mediated MC activation through the PI3K/PLCγ1 axis

**Fig. 4:**
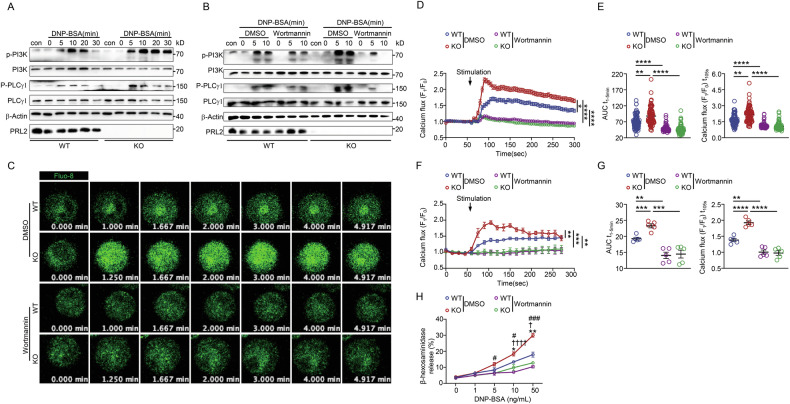
PRL2 deficiency induces mast cells activation through the PI3K/PLCγ1 axis. **A** Immunoblot images for signaling proteins in WT and KO BMMCs stimulated with IgE/DNP-BSA for the indicted times. **B** Immunoblot images for signaling proteins in WT and KO IgE primed BMMCs pre-treated with DMSO or wortmannin for 2 h, then stimulated with DNP-BSA for the indicted times. Data are representative of three independent experiments. **C**–**G** WT and KO BMMCs were sensitized with 1 μg/mL anti-DNP-IgE and loaded with Fluo-8 AM. Cells were pre-treated with DMSO or wortmannin for 2 h before stimulation with 50 ng/mL DNP-BSA. Then data was analyzed by time-lapse confocal microscope or flow cytometry. **C** Fluo-8 AM dependent measurements of cytosolic Ca^2+^ concentrations in the Tyrode buffer. Representative time-lapse of a single BMMC. The time scale reflects the kinetics of the calcium responses (Green, [Ca^2+^]_i_). **D** DNP-specific IgE sensitized WT and KO BMMCs were pretreated with DMSO or wortmannin for 2 h and then stimulated with DNP-BSA (50 ng/mL) at 1 min (arrow) using a time-lapse confocal microscope (*n* = 90 cells for WT DMSO group, *n* = 92 cells for KO DMSO group, *n* = 92 cells for WT wortmannin group, *n* = 92 cells for KO wortmannin group, ^*^P = 0.0333 for WT DMSO vs KO DMSO, ^****^P < 0.0001 for WT DMSO vs WT wortmannin, ^****^P < 0.0001 for KO DMSO vs KO wortmannin). **E** Quantitative AUC (left panel) was used to estimate the overall increase in cytoplasmic Ca^2+^ concentration over time and histogram (right panel) showing average peak cytoplasmic Ca^2+^ concentration at 105 s from (**D**). (*n* = 90 cells for WT DMSO group, *n* = 92 cells for KO DMSO group, *n* = 92 cells for WT wortmannin group, *n* = 92 cells for KO wortmannin group, ^**^P = 0.0063 and ^****^P < 0.0001 for AUC t_1-5min_, ^**^P = 0.0053 for WT DMSO vs KO DMSO, ^****^P < 0.0001 for WT DMSO vs WT wortmannin, ^****^P < 0.0001 for KO DMSO vs KO wortmannin for calcium flux (F_1_/F_0_) t_105s_). **F** DNP-specific IgE sensitized WT and KO BMMCs were pretreated with DMSO or wortmannin for 2 h and then stimulated with DNP-BSA (50 ng/mL) at 1 min (arrow) using time-lapse flow cytometry (*n* = 5 for each group, *P* = 0.0013 for WT DMSO vs KO DMSO, *P* = 0.0003 for KO DMSO vs KO wortmannin, *P* = 0.0014 for WT DMSO vs WT wortmannin). **G** Quantitative AUC (left panel) was used to estimate the overall increase in cytoplasmic Ca^2+^ concentration over time and histogram (right panel) showing average peak cytoplasmic Ca^2+^ concentration at 105 s from (**F**). (*n* = 5 for each group, ^**^P = 0.0013, ^***^P = 0.0009, ^***^P = 0.0002 for AUC t_1–5 min_, ^**^P = 0.002 and ^****^P < 0.0001 for calcium flux (F_1_/F_0_) t_105s_). **H** Dose-dependent β-hexosaminidase release in BMMCs pre-treated with DMSO or wortmannin for 2 h was measured (*n* = 3 for each group, ^*^P = 0.0205, ^**^P = 0.0037 for WT DMSO group vs KO DMSO group, ^†^*P* = 0.0138 and ^††††^*P* < 0.0001 for WT DMSO vs WT wortmannin, ^#^*P* = 0.0113, ^#^*P* = 0.0192 and ^###^*P* = 0.0002 for KO DMSO group vs KO wortmannin group). Immunoblot images (**A**, **B**) were representative of three independent experiments. Confocal images (**C**) are representative of three independent experiments. Data were obtained from three (**D**–**H**) independent experiments. Data are presented as the mean ± SEM and analyzed using Kruskal–Wallis test (**D**, **E**), two-way ANOVA (**F**), and two-tailed unpaired *t* test (**G**, **H**). ^*^P < 0.05, ^**^P < 0.01, ^***^P < 0.001, ^****^P < 0.0001.

According to the results of proteomic analysis, PLD signaling, which contributes to the signaling cascade of MC activation [22], was enriched. MC PI3K, through catalyzing the production of phosphatidylinositol 3,4,5-trisphosphate (PIP_3_) [23–25], plays a critical role in the activity of a number of MC proteins including PLD, PKC, and PLCγ. Phosphorylation of PI3K and PLCγ1 was increased following IgE/DNP-BSA stimulation of PRL2 KO BMMCs (Fig. 4A). Furthermore, when WT and KO BMMCs were pre-treated with wortmannin, a specific inhibitor of PI3K, phosphorylation of both PI3K and PLCγ1 were reduced (Fig. 4B). Inhibition of PI3K activity by wortmannin also reduced calcium (Fig. 4C–G and Video S4) and β-hexosaminidase release (Fig. 4H). Overall, these data suggest that PRL2 deficiency could promotes the MC activation through PI3K/PLCγ1 signaling.

### Inhibiting PRL2 degradation by hydroxychloroquine ameliorates passive systemic anaphylaxis

**Fig. 5:**
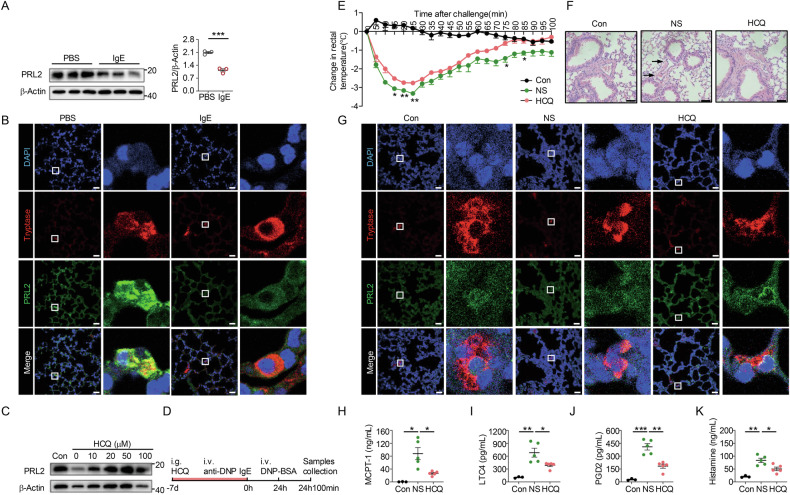
HCQ alleviated severity of passive systemic anaphylaxis by blocking PRL2 degradation. **A** Representative immunoblot images (left panel) and quantification of protein levels of PRL2 (right panel) in BMMCs with PBS or IgE stimulation for 24 h (*n* = 3 biological samples, *P* = 0.0007). **B** Representative immunofluorescence images of lung from PBS or IgE treated mice. Blue, DAPI; Red, tryptase; Green, PRL2; Scale bar, 25 μm. The area in the white box were enlarged by side. **C** Representative immunoblot images of PRL2 protein in hydroxychloroquine (HCQ) pre-treated BMMCs with indicated concentrations, and stimulated with IgE for 24 h. **D** The schematic diagram design of HCQ treatment. Mice received HCQ or normal saline (NS) by oral gavage for 7 days and passive systemic anaphylaxis (PSA) then induced, or just treated with NS and intravenously with anti-DNP-IgE as control. **E** Rectal temperatures of mice were assessed every 5 min (*n* = 3 mice for con group and *n* = 5 mice for NS and HCQ group, ^*^P < 0.05 and ^**^P < 0.01 for NS group vs HCQ group). **F** Representative images of pulmonary perivascular edema in the indicated experimental group 100 min post-challenge (black arrow: pulmonary perivascular edema). **G** Representative immunofluorescence images of lung in control, NS, and HCQ group. Blue, DAPI; Red, tryptase; Green, PRL2; Scale bar, 25 μm. The area in the white box was enlarged by side. Serum levels of MCPT-1 (**H**), LTC4 (**I**), PGD2 (**J**), and histamine (**K**) from mice were determined by ELISA (*n* = 3 mice for con group and *n* = 5 mice for NS and HCQ group, ^*^P = 0.0127 and ^*^P = 0.0117 for MCPT-1, ^**^P = 0.0059 and ^*^P = 0.0266 for LTC4, ^***^P = 0.0003 and ^**^P = 0.0010 for PGD2, ^**^P = 0.0013 and ^*^P = 0.0159 for histamine). Immunofluorescence images were representative of three to five (**B**, **G**) independent samples. Immunoblot images were representative of three (**C**) independent experiments. HE staining images were representative of three or five (**F**) independent samples. Data are presented as the mean ± SEM and analyzed using two-way ANOVA (**E**) and two-tailed unpaired *t* test (**H**–**K**). ^*^P < 0.05, ^**^P < 0.01, ^***^P < 0.001.

It has been reported that PRL2 can be degraded during inflammation [26] and we observed that the levels of this protein were decreased in BMMCs following IgE-mediated stimulation (Fig. 5A). Consistent with this observation, PRL2 expression in MC was decreased in lung tissue of IgE-treated mice (Fig. 5B). It has been reported that HCQ treatment can block PRL2 degradation [27] and we have now also observed this in BMMCs (Fig. 5C). It has also been previously been shown that HCQ treatment can alleviate allergic asthma in mice [27]. We therefore hypothesized that HCQ administration could attenuate PSA by inhibiting a reduction in PRL2. Examination of the potential relationship between HCQ and PRL2 in the IgE/DNP-BSA induced PSA model (Fig. 5D) showed that treatment of mice with HCQ alleviated the decrease in rectal temperature (Fig. 5E) and reduced pulmonary perivascular edema (Fig. 5F). Furthermore, PRL2 degradation in lung tissue was reversed after HCQ treatment (Fig. 5G). In agreement with these results, HCQ treatment reduced the levels of MCPT-1, LTC4, PGD2, and histamine in serum (Fig. 5H–K). Thus, HCQ treatment was able to block PRL2 degradation and alleviate pathology in the PSA model.

## Discussion

Anaphylaxis, a life-threatening severe systemic allergic reaction, was a revolutionary concept when described by Portier and Richet in 1902 [28]. Allergic reactions are characterized by IgE-mediated sensitization to typically innocuous proteins [29]. MC, residing in all vascularised tissues, are the primary mediators of these FcεRI-dependent responses [30]. Owing to their strategic location at barrier tissues these cells can rapidly respond to potential threats, resulting in the release of a plethora of potent immune mediators [31, 32].

Whilst basophils and eosinophils also contribute to allergic inflammation their roles are generally considered less central compared to MC. Basophils share some functional similarities with MC, such as the expression of FcεRI and the capacity to release histamine and other mediators upon IgE-dependent activation. However, they are less abundant in tissues and are primarily found in circulation, suggesting a more limited role in the immediate phases of allergic reactions [5]. Tsujimura and colleagues have suggested that basophils are primarily involved in IgG-mediated, rather than IgE-mediated, anaphylaxis [33, 34]. Eosinophils, on the other hand, are primarily involved in the later phases of allergic inflammation, characterized by tissue damage and repair. They release a distinct set of mediators, including eosinophil-derived neurotoxins, major basic proteins, and eosinophil peroxidase, which contribute to tissue remodeling and the chronicity of allergic diseases [34].

In the present study, we found that PRL2 CKO mice exhibit a more severe PSA indicating that PRL2 influences MC-mediated PSA. Although elevated levels of inflammatory mediators in the serum are not exclusively dependent on MC activation, as other myeloid cells can also release these factors, we aim to further investigate the specific effects of PRL2 on MC differentiation and function to better understand its regulatory role in this process.

The PRL family are dual-specificity phosphatases comprising three members; PRL1, PRL2, and PRL3 [12]. Since they are highly expressed in a variety of tumors and affect cell proliferation, migration, invasion, and tumor progression, they are considered both as tumor markers and as therapeutic targets [35, 36]. PRL2 is six amino acids shorter than PRL1 and PRL3 [37]. Unlike PRL1 and PRL3, PRL2 does not affect either SRC activity or p53 expression highlighting a distinct role for PRL2 [37].

PRL2 is highly expressed in immune cells, including MCs [12], and is more highly expressed in MCs compared to PRL1 and PRL3 (expression pattern of *Ptp4a2* in MCs from BioGPS database). Therefore, exploring the potential role of PRL2 in MCs is important. Our studies revealed that whilst the absence of PRL2 did not influence the differentiation of BMMCs, PRL2 KO BMMCs exhibited enhanced β-hexosaminidase release compared to WT BMMCs. Furthermore, PRL2 deficiency led to an augmented release of prostaglandin D2 (PGD2), leukotriene C4 (LTC4), and histamine in response to IgE-FcεRI stimulation. Upon stimulation, a higher proportion of KO BMMCs underwent degranulation. In line with this observation, the gene expression and protein level of pro-inflammatory cytokines and chemokines were upregulated in stimulated KO BMMCs compared to WT BMMCs. Notably, PRL2 KO BMMCs demonstrated a significantly increased secretion of IL-6, TNF-α, GM-CSF, and CCL2 compared to their WT counterparts. Thus, PRL2 in BMMCs regulates FcεRI-mediated degranulation and cytokine expression.

It is known that PRL2 controls intracellular magnesium levels [38] and that treatment with magnesium significantly reduces MC degranulation in the region with orofacial pain in a rat model [39]. These studies indicate the potential function of PRL2 in regulating intracellular ion concentrations. The significance of Ca^2+^ influx in MC activation and degranulation has been extensively established in pharmacological studies. Utilization of the Ca^2+^ chelator EGTA resulted in near-complete inhibition of FcεRI-mediated Ca^2+^ mobilization and degranulation [38, 40]. Label-free proteomic analysis of activated WT and PRL2 KO BMMCs showed that the expression of proteins involved in PLD signaling is enhanced in the absence of PRL2 and that this pathway is associated with increased intracellular calcium influx [41, 42]. We hypothesized that increased Ca^2+^ levels caused by PRL2 deficiency could result in enhanced MC activation. This hypothesis was confirmed by calcium detection using confocal microscopy and time-lapse flow cytometry.

The process of IgE-mediated degranulation involves compound exocytosis, where the membranes of adjacent granules fuse with each other and with the plasma membrane, creating intracellular degranulation chambers [43]. FcεRI-mediated MC activation triggers allergic responses by inducing rapid cell degranulation and secretion of newly synthesized cytokines and chemokines [44]. MC activation via FcεRI results in a longer time partition between signaling and secretion, associated with a period of sustained elevation of [Ca^2+^]_i_ levels [45]. We used established methods [15] to further identify the spatiotemporal pattern of degranulation caused by PRL2 deficiency. The rapid release of heterogeneous granules may reflect severe allergic reactions in vivo. Not only was there a shortened partition time but also more heterogeneously shaped granular structures. Given the critical role of SNARE proteins in the exocytotic mechanism of MCs and their potential connection to PRL2, we intended to explore the significance of SNARE complexes. VAMP-8 is the primary v-SNARE in MCs, while SNAP-23, a t-SNARE located on the plasma membrane, pairs with VAMP-8 to form the SNARE complex, which is essential for driving fusion and facilitating granule exocytosis [15, 21]. We observed a slight interaction between VAMP-8 and SNAP-23 molecules in PRL2 KO BMMCs, which may contribute to a faster degranulation process and the release of granules with more heterogeneous shapes, potentially enhancing their effect during subsequent antigen stimulation. The underlying mechanisms remain to be further investigated.

In MCs, following FcεRI aggregation, PI3K becomes activated. By catalyzing the production of phosphatidylinositol 3,4,5-trisphosphate (PIP_3_) PI3K plays a critical role in the activity of various proteins including PKC, PLCγ, and PLD [23–25, 46], thereby facilitating the signaling cascade involved in MC activation. Our mechanistic studies have shown that the PI3K pathway is involved in regulating calcium flow and degranulation in PRL2 KO BMMCs. Phosphorylation of PI3K and PLCγ1 was increased in PRL2 KO BMMCs stimulated with IgE/DNP-BSA. Pre-treatment of both WT and KO BMMCs with wortmannin, a specific PI3K inhibitor, reduced phosphorylation of PI3K and PLCγ1. Furthermore, the results showed that calcium and β-hexosaminidase release were reduced. Thus, the absence of PRL2 in BMMCs promotes the activation of PI3K which in turn triggers a series of signaling cascades ultimately leading to the release of intracellular calcium stores or the opening of calcium ion channels. This results in an increase in the concentration of intracellular calcium ions.

Finally, we observed an alteration in the severity of disease in PSA mice when PRL2 degradation was reduced. HCQ treatment reduces airway inflammation in vivo [27] and inhibits inflammation-induced PRL2 degradation [11]. HCQ was originally used as an antimalarial drug and is currently sometimes used to treat rheumatic autoimmune disorders [47] or as prophylaxis for COVID-19 [48]. Our observations suggest HCQ can attenuate PSA severity by inhibiting PRL2 degradation and thus may lead to an expanded therapeutic use of HCQ.

Nonetheless, this study had some limitations. First, we used CKO mice to detect the role of PRL2 in allergic diseases. These findings require further validation using MC-specific PRL2 KO mice. Second, whilst HCQ treatment can block the PRL2 degradation [27] it does not promote the expression and activity of PRL2. Although HCQ has already been used clinically its potential expansion to treating allergic disease needs to be assessed. Furthermore, the development of agonists for PRL2 should be explored.

**Fig. 6:**
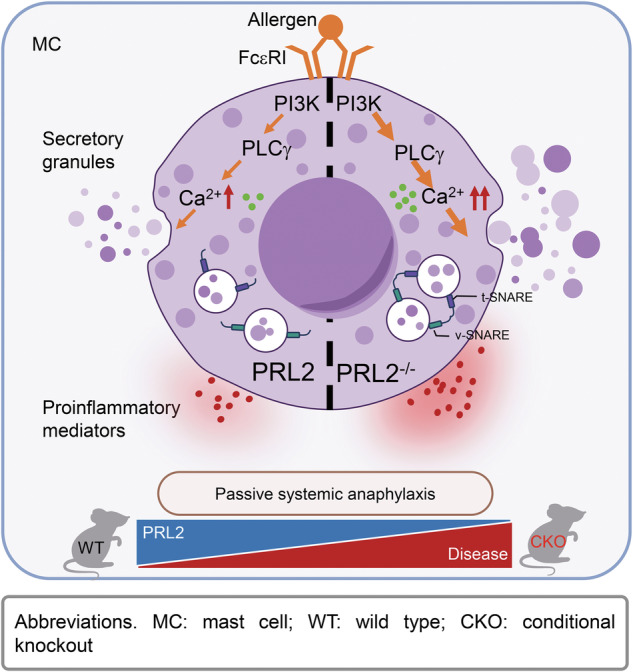
Schematic diagram of PRL2 in mast cell activation and passive systemic anaphylaxis. PRL2-deficient mast cells exhibit enhanced activation upon IgE/DNP-BSA stimulation. Intracellular calcium levels are significantly elevated via the PI3K/PLCγ pathway, leading to stronger degranulation and mediator release. The interaction between t-SNARE and v-SNARE may regulate degranulation dynamics. Additionally, PRL2 conditional knockout mice display more severe allergic reactions, suggesting that PRL2 could serve as a potential pharmacological target for the treatment of anaphylaxis.

In conclusion, our study provides new insights into the role of PRL2 in MC activation and disease progression. The cartoon mechanism figure (Fig. 6) illustrates that PRL2 modulates MC activation by regulating cytoplasmic calcium influx through the PI3K/PLCγ pathway. The interaction between t-SNARE and v-SNARE may regulate degranulation dynamics. Inhibition of PRL2 degradation by HCQ administration in WT mice decreased the severity of PSA. The results of our study indicate that PRL2 functions as an inhibitory factor in FcεRI-mediated MC activation and allergic responses. Consequently, focusing on PRL2 as a therapeutic target could represent a promising strategy for treating allergic diseases.

## Supplementary information

Supplementary information

Time-lapse flow cytometric analysis of BMMC degranulation. (Video S1 corresponds to Fig 2E).

Intracellular calcium flux dynamics in BMMCs upon stimulation with DNP-BSA. (Video S2 corresponds to Fig 3C).

Granule exocytosis was monitored using Av.SRho-binding assays (Video S3 corresponds to Fig 3H).

Intracellular calcium flux dynamics of DNP-BSA simulated BMMCs treated with wortmannin. (Video S4 corresponds to Fig 4C)

Original western blots

## Data Availability

The mass spectrometry proteomics data have been deposited to the ProteomeXchange Consortium via the PRIDE partner repository with the dataset identifier PXD053219. All data supporting this study are presented in this published article and in its supplementary information files.
