# Supplementary material for: PRL2 negatively regulates FcεRI mediated activation of mast cells
Source: Cell Death Dis. 2025 Apr 21;16(1):322. doi: 10.1038/s41419-025-07649-2 (PMC12012171; doi:10.1038/s41419-025-07649-2)
Supplement: Supplementary file 1 — Supplementary information [file 41419_2025_7649_MOESM1_ESM.docx]

Supplementary information for

**PRL2 negatively regulates FcεRI mediated activation of mast cells**

Supplementary Table 1. Primer sequences of primers used in qPCR experiment.

| *Il3* | F: 5’-GGGATACCCACCGTTTAACCA-3’ |
| --- | --- |
|  | R: 5’-AGGTTTACTCTCCGAAAGCTCTT-3’ |
| *Il4* | F: 5’-GGTCTCAACCCCCAGCTAGT-3’ |
|  | R: 5’-GCCGATGATCTCTCTCAAGTGAT-3’ |
| *Il9* | F: 5’-TGATCCACCGTCAAAATGCA-3’ |
|  | R: 5’-CCGATGGAAAACAGGCAAGA-3’ |
| *Il13* | F: 5’-CCTGGCTCTTGCTTGCCTT-3’ |
|  | R: 5’-GGTCTTGTGTGATGTTGCTCA-3’ |
| *Il6* | F: 5’-TAGTCCTTCCTACCCCAATTTCC-3’ |
|  | R: 5’-TTGGTCCTTAGCCACTCCTTC-3’ |
| *Tnfα* | F: 5’-CCCTCACACTCAGATCATCTTCT-3’ |
|  | R: 5’-GCTACGACGTGGGCTACAG-3’ |
| *Csf* | F: 5’-GGCCTTGGAAGCATGTAGAGG-3’ |
|  | R: 5’-GGAGAACTCGTTAGAGACGACTT-3’ |
| *Ccl1* | F: 5’-GGCTGCCGTGTGGATACAG-3’ |
|  | R: 5’-AGGTGATTTTGAACCCACGTTT-3’ |
| *Ccl2* | F: 5’-TTAAAAACCTGGATCGGAACCAA-3’ |
|  | R: 5’-GCATTAGCTTCAGATTTACGGGT-3’ |
| *Ccl3* | F: 5’-TTCTCTGTACCATGACACTCTGC-3’ |
|  | R: 5’-CGTGGAATCTTCCGGCTGTAG-3’ |
| *Actb* | F: 5’-GGCTGTATTCCCCTCCATCG-3’ |
|  | R: 5’-CCAGTTGGTAACAATGCCATGT-3’ |





Fig S1. PRL2 deficiency does not affect cells proportion in peripheral blood.

The proportion of lymphocytes, monocytes, neutrophils, eosinophils and basophils was assessed by Wright-Giemsa staining. One hundred white cells were counted at high magnification to distinguish above cells. Data were obtained from four independent samples.

Data are presented as mean ± SEM and analyzed using two-tailed unpaired *t* test.





Fig S2. PRL2 deficiency does not affect BMMC differentiation.

(A) The representative images of mature BMMC purity (left panel) and statistical analysis of BMMC purity during culture (right panel) (n=3 biological samples for each group).

(B) Protein levels of PRL2 in BMMCs were analyzed by western blot.

(C) The representative images (left panel) and statistical analysis (right panel) of mature BMMC proliferation with indicated concentrations of stem cell factor (SCF) for 72h.

Representative flow plots (A) and Immunoblot images (B) were representative of three experiments. Representative flow plots of CFSE-labeled BMMCs (C) gated on CD117^+^FcεRI^+^ cells were representative of five experiments. Data are presented as mean ± SEM and analyzed using two-tailed paired *t* test (A, C).





Fig S3. Over-expression of PRL2 decreases IgE-mediated degranulation and cytokines expression

(A) Protein levels of PRL2 in KO BMMCs replenished with Ctrl and PRL2-MigR1.

(B-D) KO and over-expression of PRL2 BMMCs were sensitized with anti-DNP-IgE and stimulated with DNP-BSA for indicated time.

(B) BMMCs were activated with indicated concentrations of DNP-BSA for 30min and dose-dependent β-hexosaminidase release was measured (n=four for each group, ^**^*P=*0.0013).

(C) Degranulation was assessed by measuring the production of LTC4 and histamine at 6h (n=4 for each group, *P=*0.0012 for LTC4, *P=*0.0056 for histamine).

(D) *IL-4* and *CCL2* gene expression of BMMCs, which replenished with Ctrl and PRL2-MigR1, stimulated with 50ng/mL DNP-BSA for indicated time were analyzed by qPCR, normalized to WT control (Con) group (n=4 for each group, ^**^*P=*0.0010 and ^*^*P=*0.0430 for IL4, *P=*0.0487 for CCL2).

Immunoblot images (A) were representative of three experiments. Data are presented as mean ± SEM and analyzed using two-tailed unpaired *t* test (B-D).





Fig S4. PRL2 deficiency affects SNARE complex formation.

WT and KO BMMCs were sensitized with anti-DNP-IgE and stimulated with DNP-BSA for indicated time. Immunoprecipitation of SNAP-23/VAMP-8 complexes in resting BMMCs (Con) or activated with DNP-BSA for indicated time. Immunoprecipitated SNAP-23 was resolved and probed for VAMP-8.

Video S1. Time-lapse flow cytometric analysis of BMMC degranulation. (Video S1 corresponds to Fig 2E).

Time-lapse flow cytometry analysis of WT (left) and KO (right) BMMC degranulation. Anti-DNP-IgE-sensitized BMMCs stimulated with 50 ng/mL DNP-BSA (added at t=1 min) in the presence of Av.SRho were inspected using the time-lapse function of a BD LSRFortessa cytometer. Image sequences of the time-lapse recordings were processed using the Fiji software.

Video S2. Intracellular calcium flux dynamics in BMMCs upon stimulation with DNP-BSA. (Video S2 corresponds to Fig 3C).

Anti-DNP-IgE-sensitized WT (left) and KO (right) BMMCs were loaded with Fluo-8 AM and stimulated with DNP-BSA. Fluo-8 (green, [Ca^2+^]_i_) fluorescence signals were monitored at the single cell level using time-lapse confocal microscopy in a controlled atmosphere (37℃ and 5% CO_2_).

Video S3. Granule exocytosis was monitored using Av.SRho-binding assays (Video S3 corresponds to Fig 3H).

Anti-DNP-IgE-sensitized WT (left) and KO (right) BMMCs were loaded with Fluo-8 AM and stimulated with DNP-BSA in the presence of Av.SRho. Fluo-8 (green, [Ca^2+^]_i_) and Av.SRho (red) fluorescence signals were monitored at the single cell level using time-lapse confocal microscopy in a controlled atmosphere (37℃ and 5% CO_2_).

Video S4. Intracellular calcium flux dynamics of DNP-BSA simulated BMMCs treated with wortmannin. (Video S4 corresponds to Fig 4C)

Anti-DNP-IgE-sensitized WT (left) and KO (right) BMMCs were pretreated with DMSO or wortmannin for 2h, then loaded with Fluo-8 AM and stimulated with DNP-BSA. Fluo-8 (green, [Ca^2+^]_i_) fluorescence signals were monitored at the single cell level using time-lapse confocal microscopy in a controlled atmosphere (37℃ and 5% CO_2_).
