# Supplementary material for: PRL2 negatively regulates FcεRI mediated activation of mast cells
Source: Cell Death Dis. 2025 Apr 21;16(1):322. doi: 10.1038/s41419-025-07649-2 (PMC12012171; doi:10.1038/s41419-025-07649-2)

Figure 4

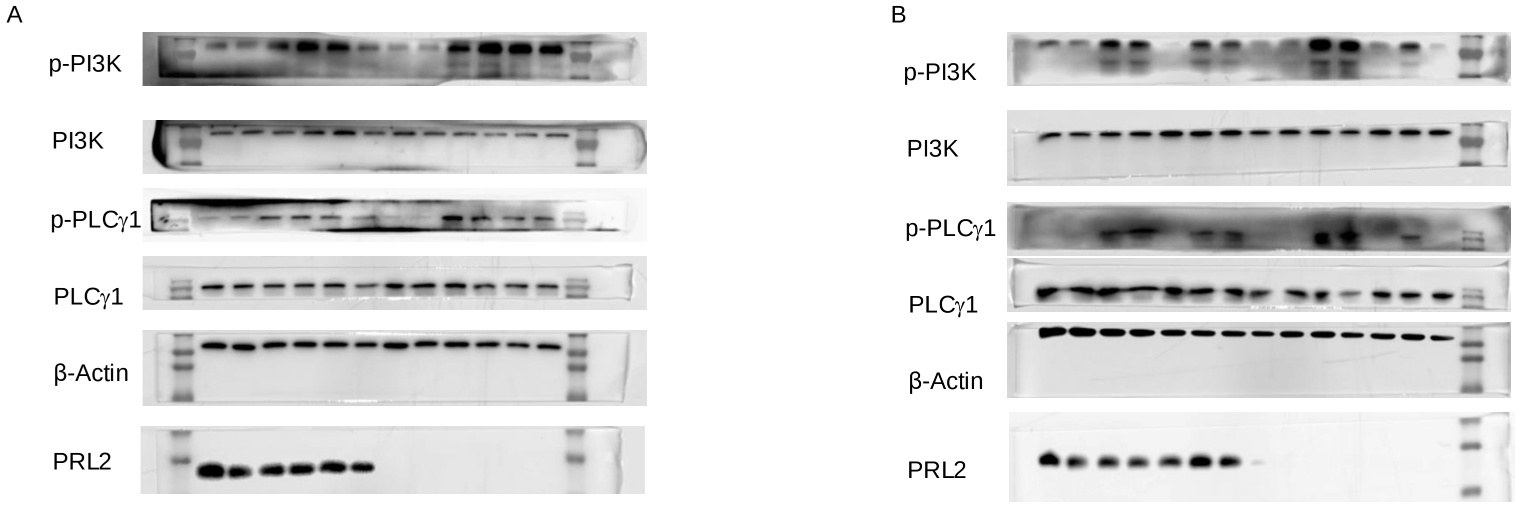

A
p-PI3K
PI3K
p-PLCγ1
PLCγ1
β-Actin
PRL2
B
p-PI3K
PI3K
p-PLCγ1
PLCγ1
β-Actin
PRL2

Figure 5

A

PRL2

β-Actin

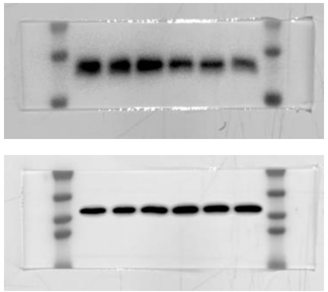

C

PRL2

β-Actir

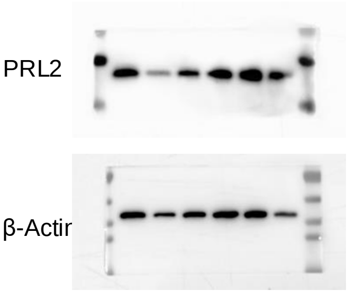

Supplementary Figure 2

B

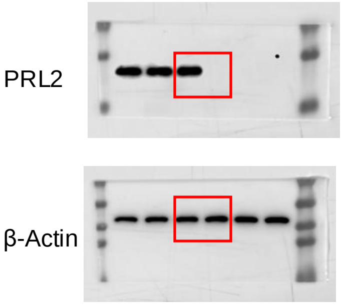

Supplementary Figure 3

A

PRL2

β-Actin

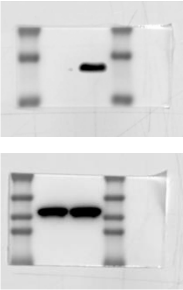

Supplementary Figure 4

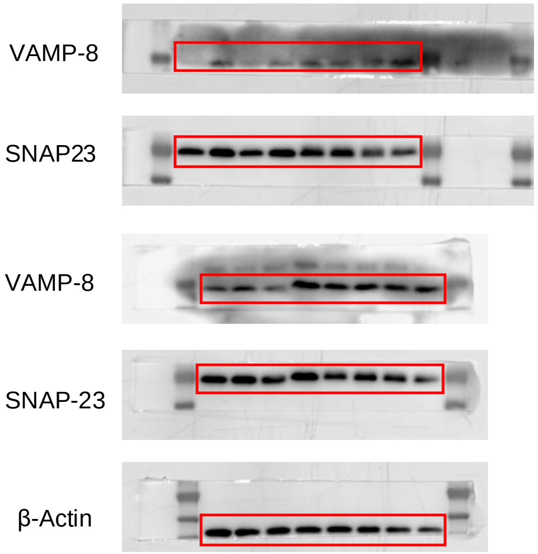

Supplement: Supplementary file 6 — Original western blots [file 41419_2025_7649_MOESM6_ESM.pdf]
